# Source credibility modulates the validation of implausible information

**DOI:** 10.3758/s13421-020-01067-9

**Published:** 2020-07-10

**Authors:** Andreas G. Wertgen, Tobias Richter

**Affiliations:** Department of Psychology IV, University of Würzburg, Röntgenring 10, 97070 Würzburg, Germany

**Keywords:** Validation, Plausibility, Sourcing, Credibility, Text comprehension

## Abstract

Validation of text information as a general mechanism for detecting inconsistent or false information is an integral part of text comprehension. This study examined how the credibility of the information source affects validation processes. Two experiments investigated combined effects of source credibility and plausibility of information during validation with explicit (ratings) and implicit (reading times) measurements. Participants read short stories with a high-credible versus low-credible person that stated a consistent or inconsistent assertion with general world knowledge. Ratings of plausibility and ratings of source credibility were lower when a credible source stated a world-knowledge inconsistent assertion compared with a low-credible source. Reading times on target sentences and on spillover sentences were slower when a credible source stated an assertion inconsistent with world knowledge compared with a low-credible source, suggesting that source information modulated the validation of implausible information. These results show that source credibility modulates validation and suggest a bidirectional relationship of perceived plausibility and source credibility in the reading process.

When readers read a text for comprehension, they continually build a mental representation of the situation described in the text (e.g., persons, events, actions, state of affairs). This type of representation is called the situation model (van Dijk & Kintsch, 1983; Zwaan & Radvansky, 1998) or mental model (Johnson-Laird, 1983). The construction of situation models during comprehension entails a mechanism of validation, that is, an evaluation of the plausibility of incoming information by determining its fit with the current situation model and accessible world knowledge (Richter, 2015). Various experimental approaches have provided evidence that readers continually evaluate text information based on activated world knowledge and contextual information (e.g., Cook & O’Brien, 2014; O’Brien & Cook, 2016; Richter, Schroeder, & Wöhrmann, 2009; Singer, 2013). A strong body of evidence has accumulated for validation as a routine process and its importance for text comprehension (e.g., Ferretti, Singer, & Patterson, 2008; Maier & Richter, 2013; Rapp & Kendeou, 2009; Schroeder, Richter, & Hoever, 2008). However, research systematically mapping out the conditions that affect validation is fairly new. The conditions examined to date include the contributions of world knowledge and contextual information (e.g., Isberner & Richter, 2014; van Moort, Koornneef, & van den Broek, 2018; Walsh, Cook & O’Brien, 2018; Williams, Cook, & O’Brien, 2018), individual differences in working memory capacity and access to world knowledge (Singer & Doering, 2014), individual differences in beliefs (Gilead, Selal, & Marid, 2018), developmental influences (Piest, Isberner, & Richter, 2018), text genre (such as fantasy text: Creer, Cook, & O’Brien, 2018), and recency of text information (Guéraud, Walsh, Cook, & O’Brien, 2018).

One specific type of contextual information that might affect validation processes is the credibility of the source that provides the information. Information about source credibility can signal to the reader whether information provided by the source is believable and thus bears a strong conceptual relationship to the validity of information. Do readers consider source credibility during the validation of text information, and if they do, how does source credibility affect validation? This question is theoretically and practically relevant. A number of studies suggest that readers sometimes fall prey to obviously false information embedded in fictional narratives, such as “The Atlantic is the largest ocean,” and then use this information in later knowledge tests, although they know in principle that it is false (e.g., Marsh & Fazio, 2006; Rapp, 2008). Besides the plausibility of information, the credibility of the information source (e.g., a character who makes a statement) is a relevant cue that readers might rely on to guard themselves against misinformation. The extent that readers use this cue during validation is an open question. At a general level, the present study contributes to the question of how contextual information (e.g., discourse knowledge) and world knowledge are used and potentially compete against each other during text processing, an issue which has been highlighted in the RI-Val model (O’Brien & Cook, 2016).

Methodologically, two basic approaches are used to investigate comprehension processes such as validation. Researchers can investigate online processes during comprehension with implicit measurements such as reading times, which are informative with regard to moment-to-moment comprehension processes. Alternatively, researchers can ask specific questions or prompt readers to judge certain characteristics of the text, which are potentially very informative but must be collected off-line (i.e., after reading), which limits their value for studying comprehension processes. An approach using both explicit (off-line) judgments and implicit (online) measures allows examining their convergences and divergences (Rapp & Mensink, 2011). This approach offers a more complete picture of the nature of the processes involved in comprehension and a way to better understand the meaning of reading times that are notoriously ambiguous even in the context of validation.

The two experiments presented in this paper used short narratives and explicit and implicit measures of validation to test the hypothesis of an interactive effect of information plausibility and source credibility on validation. Both experiments used implausible information that was clearly inconsistent with general world knowledge (similar to Marsh & Fazio, 2006; Rapp, 2008). In the following section, we briefly discuss research on validation during comprehension, followed by a review of studies that have examined the role of source credibility on text comprehension. Finally, to explain the background of the hypotheses tested in the two experiments, we discuss in detail the small body of extant studies that have examined combined effects of plausibility and source credibility.

## Validation: Assessing the plausibility of information

Evidence has accumulated showing that readers routinely assess the plausibility of information during reading. Plausibility can be defined as the “acceptability or likelihood of a situation or a sentence describing it” (Matsuki et al., 2011, p. 926) or as “the degree of fit between a given scenario and prior knowledge” (Connell & Keane, 2006, p. 98). Consistent with these two definitions, plausibility can be seen as an assertion that varies along a continuum with true and false representing its end points.

Richter et al. (2009) introduced the epistemic Stroop paradigm, which aims at unravelling the nonstrategic, routine character of validation. The underlying logic of this paradigm is that reading a true (plausible) or false (implausible) sentence with regard to world knowledge should elicit an automatic response tendency depending on the plausibility of the information. This response tendency should interfere with an unrelated task, much like the interference effect underlying the original color-naming task invented by Stroop (1935). Several studies have found such epistemic Stroop effects with different stimuli and tasks. Richter et al. (2009) found this interaction pattern in two experiments with true versus false statements. Further experiments have yielded epistemic Stroop effects for assertions of varying plausibility (Isberner & Richter, 2013) with belief-consistent and belief-inconsistent statements (Gilead et al., 2018), a nonlinguistic task (judging the color of a word; Isberner & Richter, 2013), a nonevaluative probe task (Isberner & Richter, 2014), and audiovisual information (Piest et al., 2018). These studies provide broad evidence for validation as a nonstrategic, involuntary process and for the assumption that validation produces implicit plausibility judgments and are more than mere disruptions of comprehension.

Further evidence for routine validation comes from experiments based on eye tracking (Matsuki et al., 2011), event-related potential data (e.g., Ferretti et al., 2008), and reading times (e.g., Cook & O’Brien, 2014). For example, a typical finding from numerous experiments with the so-called inconsistency paradigm is that reading times are longer for sentences that conflict with information provided earlier in the text and pertinent prior knowledge (e.g., O’Brien, Rizzella, Albrecht, & Halleran, 1998). Singer (2006) showed that the pattern of the reading time for true versus false affirmative and negated sentences mirrors the pattern in explicit verification judgments.

O’Brien and Cook (2016) proposed the resonance-integration-validation model (RI-Val), a comprehensive theory of comprehension in which validation plays a prominent role as pattern-matching process. This model assumes three types of processes: resonance, integration, and validation that are relevant for establishing a coherent representation during reading. All three processes are assumed to be passive, parallel and nonstrategic, and asynchronous but overlapping, and they are assumed to run to completion. Incoming text information activates background knowledge (e.g., discourse and world knowledge) through a resonance-like process (cf. Myers & O’Brien, 1998; O’Brien & Myers, 1999). After a certain amount of knowledge has been activated through this resonance process (*R*), the next process of integrating the activated knowledge with the text information (*I*) begins. After integration has reached a sufficient conceptual overlap, the validation process (*Val*) begins by evaluating the activated, integrated information against activated relevant background knowledge. When validation has reached a sufficient level, called the coherence threshold, the reader can then process subsequent text information. The parallel but asynchronous fashion of activation, integration, and validation is a distinct assumption of the RI-Val model (Cook, 2014; Cook & O’Brien, 2014). The assumption implies that validation processes can take effect with a delay, such as a slowdown of reading at a spillover sentence following a critical sentence that conveys implausible information.

Recent studies have investigated these critical assumptions of the RI-Val model and influencing conditions of validation, such as the competition of contextual information versus world knowledge or recency of information. When reading fantasy texts, readers are confronted with violations of real-world knowledge, yet they seem to have no comprehension difficulties. Walsh et al. (2018) investigated which source of information dominates validation with either fantasy-unrelated or fantasy-related inconsistencies in an extended fantasy narrative. Their experiments show that contextual information and world knowledge compete, but even if contextual information initially dominates validation, world knowledge can still influence comprehension. Using short texts about correct or incorrect historical events, van Moort et al. (2018) found distinct differences in text-based and knowledge-based monitoring that were biased by a context leading towards a correct or an incorrect event. Although contextual information and world knowledge had an effect on reading times of target sentences, only inconsistencies in world knowledge elicited spillover effects. Williams et al. (2018) investigated incomplete validation with semantic illusions (e.g., Moses illusion; Erickson & Mattson, 1981) embedded in narratives with varying contextual support, showing that both general world knowledge and contextual information can be (re)activated and influence comprehension. More importantly, their study presents evidence that readers are consistently disrupted by semantic illusions, even when semantic illusions are undetected.

To conclude, a growing body of empirical evidence suggests different time courses for integration and validation of contextual information and world knowledge. In line with the RI-Val model, both sources of information can influence validation. Effects of validation often occur at a delay—that is, at a spillover sentence following the critical information, which is in line with the idea of activation, integration, and validation as parallel but asynchronous processes.

## Evaluation of source credibility

The credibility of an information source may be construed as a type of contextual information that bears a specific relationship to validation. Source credibility can depend on a variety of aspects associated with the communicator. Most conceptualizations of source credibility address the two dimensions of expertise and trustworthiness (Lombardi, Seyranian, & Sinatra, 2014; Self, 2009). Our experiments focused on the expertise aspect of source credibility. Expertise in this context “refers to the extent to which a speaker is received to be capable of making correct assertions” (Pornpitakpan, 2004, p. 244). Evidence for the relevance of source credibility for text comprehension comes from research on the comprehension of multiple texts on the same topic (e.g., documents on a historical event, scientific texts dealing with the same phenomenon, argumentative texts discussing the same political issue) from different perspectives (e.g., Bråten & Braasch, 2018). In multiple text comprehension, source characteristics (e.g., text type, author, language style) can be used as the basis for evaluations of source credibility, which is especially important to make sense of multiple texts with conflicting information. For example, Bråten, Stromso, and Britt (2009) found an effect of source trustworthiness ratings on comprehension of multiple texts about climate change. Steffens, Britt, Braasch, Stromso, and Bråten (2014) found less recall for low-credible sources (e.g., sources overstating results) than sources that presented evidence appropriately, showing to some extent a memory effect of source credibility. In sum, source credibility is recognized as an important variable in multiple text comprehension and the broader field of how people interact with information on the internet (e.g., Wathen & Burkell, 2002). However, relatively few studies have examined the role of source credibility in understanding information in single texts and its effects on comprehension processes.

## Evaluation of plausibility and source credibility

To date, few studies have examined the combined effects of source credibility and plausibility on validation and comprehension. Overall, the findings of this research are inconclusive. In Sparks and Rapp (2011), participants read interview transcripts in four reading-time experiments in which information about a character was provided by the interviewed person who was described as a credible (honest and trustworthy) or noncredible (dishonest and untrustworthy) source. This character was described with a specific trait that could be inferred from the text (e.g., being messy). Source descriptions varied in the trustworthiness ascribed to the source, whereas source expertise was held constant. Later in the texts, the reader learned whether the protagonist who was introduced in the beginning was behaving in a manner that was either trait consistent or inconsistent. The results of Experiments 1 to 3 indicated little influence of source credibility at the encoding level. Only when participants were instructed to explicitly judge the likelihood of future character behaviors, source credibility significantly affected other processing stages. Sparks and Rapp (2011) concluded that source credibility can influence text comprehension, but only under specific circumstances—for example, when readers follow a specific reading goal.

Braasch, Rouet, Vibert, and Britt (2012) provided source information within the text. Braasch and colleagues conducted studies that focused on plausibility and source credibility in their investigation of the discrepancy-induced source comprehension (D-ISC assumption), which builds on the documents model framework. The D-ISC assumption holds that when readers encounter discrepant (i.e., inconsistent) information in a text, they become more attentive to sources, possibly in an attempt to resolve the discrepancy. To test this assumption, Braasch et al. used brief news articles (two sentences) in an eye-tracking study, which presented two sources (e.g., an art critic versus a lighting technician) that made claims about various topics (e.g., an opera show). The claims were either consistent or discrepant. Participants who summarized texts with discrepant information reported more sources, had better memory for discrepant versions, fixated source information more often, and spent more time on source information. The basic idea of the D-ISC has been supported by a number of studies with implausible (belief-inconsistent) information (e.g., Bråten, Salmerón, & Strømsø, 2016; de Pereyra, Britt, Braasch, & Rouet, 2014) and discrepant information (e.g., discrepant claims;, Kammerer, Kalbfell, & Gerjets, 2016; Rouet, Bigot, de Pereyra, & Britt, 2016). These studies used different types of (single) texts, such as news reports or argumentative texts, and sometimes studied inconsistencies across multiple texts (e.g., Barzilai & Eshet-Alkalai, 2015; Kammerer & Gerjets, 2014; Strømsø, Bråten, Britt, & Ferguson, 2013).

Common to all of the studies on the D-ISC assumption is that they studied how discrepant or implausible information affects the processing of source information. In contrast, the present research sought to answer the question of how source information, particularly source credibility, affects the processing of implausible information (i.e., inconsistent information with general world knowledge). To our knowledge, Foy, LoCasto, Briner, and Dyar (2017) were the first to address this question by investigating a proposed interactive effect between plausibility of information and source credibility. They conducted experiments with short narratives that included implausible assertions (Experiment 1) or plausible assertions (Experiment 2) to shed light on a possible interplay between plausibility of text information and the credibility of its source. In the narratives used in Experiment 1, a trustworthy person (e.g., a sober person at a party) or an untrustworthy person (e.g., a person on drugs) stated an implausible assertion (e.g., that there are wolves in the yard). The stories continued with information that was either consistent or inconsistent with the implausible assertions. For example, a consistent continuation was a credible (sober) person who confirmed seeing wolves in the yard, whereas an inconsistent continuation was the credible person seeing just a few friends hanging out in the yard. Foy et al. argued that readers validate the implausible assertion but consider source credibility in this process. In line with this assumption, reading times of the implausible assertions, and especially the subsequent (spillover) sentence, were shorter when the assertions came from a trustworthy compared with an untrustworthy source. In contrast, reading times were shorter for consistent compared with inconsistent continuations in stories with trustworthy sources, whereas the pattern was reversed for untrustworthy sources. These results suggest that readers factored source credibility into validating implausible assertions in text narratives. Apparently, a trustworthy source can make an implausible assertion appear more plausible, leading to a less severe disruption of text comprehension. Moreover, a trustworthy source can promote the acceptance of information and its integration into the situation model, which critically hinges on the outcome of the validation process (Schroeder et al., 2008). However, a slightly different pattern emerged when plausible assertions were used in Experiment 2 of Foy et al. (2017). Although plausible assertions were read faster when the source was a trustworthy compared with an untrustworthy source, no effects were found on the spillover sentence. In terms of the RI-Val model (O’Brien & Cook, 2016), this finding indicates a faster completion of the validation process for plausible sentences. Moreover, consistent continuations were always read faster than were inconsistent continuations, suggesting that the plausible information was likely to be accepted and integrated in the situation model, regardless of source credibility. In sum, the experiments by Foy et al. (2017) show that message plausibility and source credibility each affect validation, but not in the same way. The effect of message plausibility seems to exert somewhat stronger effects, and it appears to affect the time course of validation. However, given the potential importance of source information for theories of validation, further research on the role of source credibility in validation seems warranted.

## Rationale of the present experiments

The present research aimed at examining how source credibility is considered in validation during comprehension. We used a strong manipulation of plausibility, contrasting highly implausible sentences that are inconsistent with general world knowledge (e.g., “The Atlantic is the biggest ocean in the world”) and highly plausible sentences that are consistent with general world knowledge (e.g., “The Pacific is the biggest ocean in the world”; similar to Marsh & Fazio, 2006; Rapp, 2008). These assertions were embedded in short stories and stated by a person described as a source with a high or low level of expertise. Thus, we manipulated a different facet of source credibility than Foy et al. (2017), who focused on the trustworthiness of sources. Third, we included online measures (reading times), but also off-line measures (plausibility judgments and source credibility judgments). This last part of the method provided a way to investigate possible convergences and discrepancies in moment-to-moment processes during reading and more global judgments after reading (Rapp & Mensink, 2011).

The general assumption was that textual information about source credibility, such as the expertise of a person, would affect the validation of the plausibility of the target statements. We conducted two experiments to gain a better understanding of the interplay between validation and source evaluation. Experiment 1 was based on explicit measures (plausibility and source credibility ratings), and Experiment 2 was based on implicit measures (reading times on target sentences and on spillover sentences). By including reading times for the spillover sentence in Experiment 2, we were able to further elucidate the time course of the combined effects of source credibility and plausibility in light of the RI-Val model proposed by O’Brien and Cook (2016). If source credibility is used in validation, the effects should also occur in the reading times for the spillover sentence, possibly even in a more pronounced fashion.

In the two experiments, the implausible assertions were clearly false, and the plausible statements were clearly true (i.e., consistent with world knowledge; assertions were located close to the end points of the plausibility continuum). Based on this stronger manipulation of plausibility, we expected a different pattern for the combined effect of plausibility and source credibility than Foy et al. (2017). In particular, we expected a highly implausible assertion (i.e., inconsistent with world knowledge) from a credible source to create an inconsistency at the discourse level, which should exacerbate (rather than mitigate) the disruption caused by the validation process and even increase its implausibility.

In Experiment 1, we expected readers to rate plausibility higher for assertions consistent with world knowledge (e.g., “Jupiter is the biggest planet in the solar system”) than for assertions inconsistent with world knowledge (e.g., “The sun is the biggest planet in the solar system”; Hypothesis 1). However, we also expected an interaction of plausibility and source credibility to emerge. For assertions inconsistent with world knowledge, a low-expertise source (e.g., a protagonist knowing almost nothing about astronomy and stars) should lead to higher plausibility ratings than a high-expertise source (e.g., a protagonist knowing very much about astronomy and stars), whereas the opposite pattern should occur for assertions consistent with world knowledge (Hypothesis 2).

We used the source credibility ratings obtained in Experiment 1 to explore whether plausibility also alters the perceived credibility of the source in Experiment 2. Generally, high-expertise sources should be rated as more credible than low-expertise sources. A test of this assumption can be seen as a kind of manipulation check for the source credibility manipulation. However, readers might evaluate source credibility not only based on source characteristics in the text, such as a person being described as a physics professor, but also based on the plausibility of the assertion stated by that person. Reading about a person who makes a false statement might cause readers to judge this person as less credible, regardless of the expertise level (Slater & Rouner, 1996). Finally, we assessed ratings of meaningfulness and comprehensibility for every story version to explore how these global judgments would depend on plausibility and source credibility. More importantly, the ratings of meaningfulness and comprehensibility were used to control for differences in these variables between the texts in the analyses of plausibility and source-credibility ratings in Experiment 1 and reading times in Experiment 2.

## Experiment 1

Experiment 1 investigated the effects of source credibility and plausibility on explicit ratings of plausibility and source credibility. We expected a main effect of plausibility (Hypothesis 1) on plausibility judgments, and we expected the effect of plausibility to be modulated by source credibility (Hypothesis 2). Additionally, readers evaluated the credibility of the source and rated comprehensibility and meaningfulness of the stories.

### Method

#### Participants

Sixty-seven undergraduates at the University of Würzburg (Germany) participated in this study. The mean age was 22.48 years (*SD* = 6.71). Most participants were female (77%). The data from four participants, who spoke a first language other than German, were excluded from the analyses. Sixty-five participants received study credit, and two participants received a monetary compensation (5 euros) for participation.

#### Materials

We created 36 short stories about situations from everyday life (e.g., vacations or restaurant visits; see Table 1 for an example). Each story consisted of eight sentences. The first two sentences served as an introduction. The third sentence described the protagonist either as a source with high credibility (a person with high expertise in a certain field, e.g., a mineralogist) or with low credibility (a person with low expertise, e.g., a pool attendant). The descriptions of expertise were explicit statements about the amount of expertise in a field and included other information—for example, about the profession, occupation, or academic title. The sixth sentence was the target sentence, which was an assertion stated by the person introduced in the third sentence. The assertion could be consistent (i.e., true) or inconsistent (i.e., false) with general world knowledge—for example, “That’s the Indian/Pacific Ocean, and it is between Africa and Australia.” The world-knowledge consistent and world-knowledge inconsistent assertions were partly based on available general world knowledge norms (Nelson & Narens, 1980; Tauber, Dunlosky, Rawson, Rhodes, & Sitzman, 2013) and were extended with additional statements.

**Table 1.** Sample experimental story for Experiments 1 and 2

*Introduction:* Sandra was visiting the planetarium in Bochum with her children, Eva and Torben. Both of them were very curious and had a drive to learn.	
*Expertise*	
*Low expertise:*	
Sandra had almost no knowledge about astronomy and stars.	
*High expertise:* Sandra had a lot of knowledge about astronomy and stars.	
*Continuation:* Because of that, she thought visiting a planetarium would be a great idea. On the way, Sandra told her children what they could expect.	
*Assertion*	
*World-knowledge consistent assertion:* “Jupiter is the biggest planet in the solar system,” she said.	
*World-knowledge inconsistent assertion:* “The sun is the biggest planet in the solar system,” she said.	
*Spillover:* Eva and Torben were thrilled to get to know more. *Ending:* Sandra, Eva, and Torben stayed the whole day at the planetarium.	

The possible combinations of source credibility and assertions about world knowledge yielded four story versions, two consistent (source with high expertise and world-knowledge consistent assertion, source with low expertise and world-knowledge inconsistent assertion) and two inconsistent versions (source with high credibility and world-knowledge inconsistent assertion, source with low credibility and world-knowledge consistent assertion). The seventh (spillover sentence) and eighth sentences continued the story. The stories had an average Flesch score (Flesch, 1948; German adaptation by Amstad, 1978) of 56.46 (*SD* = 5.84), which translates to “demanding” or “fairly difficult” to read.

#### Design

The design was a 2 (source credibility: high versus low expertise) × 2 (plausibility: world-knowledge consistent versus world-knowledge inconsistent assertion) within-subjects design. Half of the participants provided plausibility ratings for the target sentence, and the other half provided ratings of source credibility for the protagonist introduced in the third sentence. All participants provided ratings of meaningfulness and comprehensibility for each story. A Latin square with four different lists was used to counterbalance the assignment of stories to experimental conditions across participants.

#### Procedure

The experiment was programmed and presented with the experimenter software Inquisit 5 (Version 5; Millisecond Software, Seattle, WA). We instructed the participants to read the stories carefully and to rate either plausibility of the stated assertions or the source credibility of a described source and meaningfulness and comprehensibility of the stories. Participants read the stories on a computer screen sentence by sentence in a self-paced fashion. They were tested in groups up to four and gave informed consent before the experiment started. A fixation cross at the location of the first word was displayed for 500 ms. Participants could advance to the next sentence by pressing the space bar. Four practice trials were included at the beginning of the experiment to familiarize participants with the self-paced reading method. Letters in all sentences except the currently read one were masked with an “X.” Participants read the stories in a randomized order. Every participant could see every story in only one of the possible versions. The procedure differed depending on which of the two rating tasks participants were assigned to. Participants rated the source credibility of the protagonist after reading a story in the self-paced fashion. The story was presented again, but this time with all sentences displayed at once and with the critical sentences (three and six) highlighted in blue. Below the text, the following question was presented: “How would you judge the credibility of the person (highlighted in blue) as an information source regarding that topic?” Participants rated source credibility on a scale from 1 (*not credible at all*) to 7 (*very credible*). Participants assigned to the plausibility rating saw the question “How would you judge the plausibility of this assertion?” after they had read the sixth sentence (target sentence) and continued reading the story afterwards. They rated plausibility on a scale from 1 (*not plausible at all*) to 7 (*very plausible*). In addition, all participants rated the meaningfulness and comprehensibility of the story on 7-point scales. The experiment lasted 30 min. On average, participants needed 24.17 min (*SD* = 3.47 min) to read and rate all 36 stories.

### Results and discussion

We excluded data for one story because of one incorrectly presented version. The remaining 35 stories received satisfactory ratings for meaningfulness (*M* = 5.97, *SD* = 1.23) and comprehensibility (*M* = 5.24, *SD* = 1.75).

We estimated linear mixed models with the lmer function of the R package lme4 (Version 1.1-17; Bates, Mächler, Bolker, & Walker, 2015) for all linear mixed models (Baayen, Davidson, & Bates, 2008) and the lsmeans function in the lsmeans package (Lenth, 2016) to further analyze interactions. The Type I error probability was set at .05 (two-tailed) in all significance tests. We estimated effect sizes (Cohen’s *d*) for differences in condition means based on the approximate formula proposed by Westfall, Kenny, and Judd (2014) for linear mixed models with contrast codes and single-degree-of-freedom tests (see also Judd, Westfall, & Kenny, 2017).

Participants and stories were entered as random effects (random intercepts) in the models. The two independent variables were contrast coded, and their main effects and their interaction were entered as fixed effects in the models. Sources with high credibility (high expertise) were coded as 1, and sources with low credibility (low expertise) were coded as −1. Assertions consistent with world knowledge (high plausibility) were coded as 1 and assertions inconsistent with world knowledge (low plausibility) were coded as −1. The position of a story in the experiment was entered as centered metric predictor in the model. The incentive type (course credit or money) did not affect the results, which remained intact when the type of incentive was statistically controlled in the models.

#### Plausibility ratings

**Fig. 1:**
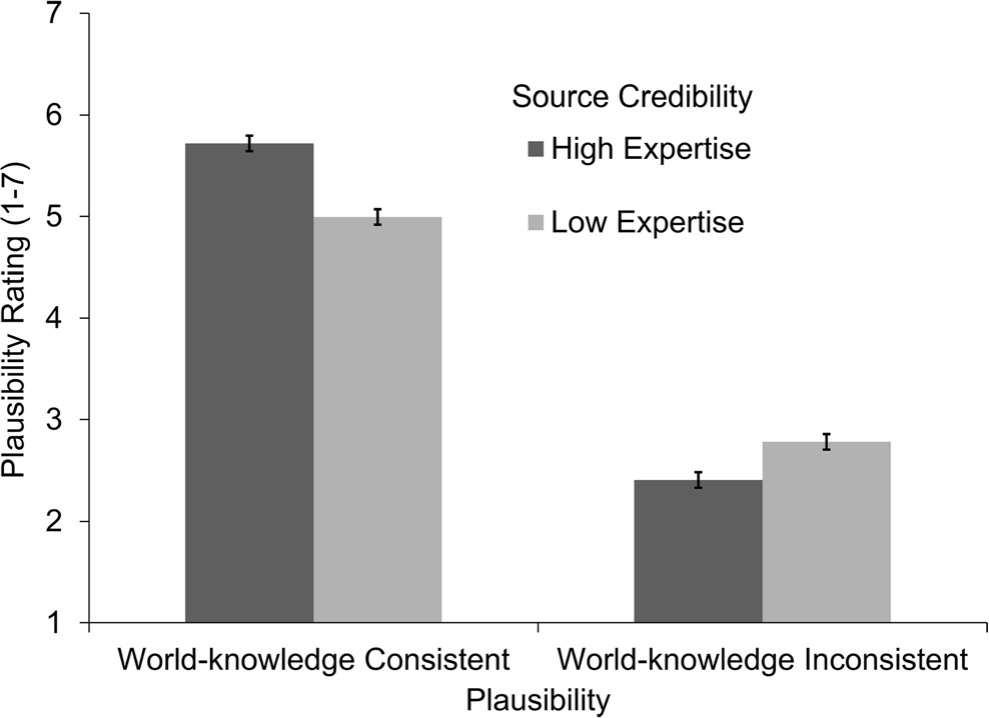
Mean plausibility ratings by experimental condition. Error bars represent the standard error of the mean

Plausibility ratings were available from 34 participants. We expected readers to rate the plausibility of assertions consistent with world knowledge higher than that of world-knowledge inconsistent assertions (Hypothesis 1). As expected, the analysis revealed a strong main effect of plausibility, β = 1.40, *t*(1,078) = 27.03, *p* < .001, *d* = 1.50. World-knowledge consistent assertions led to higher plausibility ratings (*M* = 5.36, *SE* = 0.13) than assertions inconsistent with world knowledge (*M* = 2.56, *SE* = 0.13). Analysis also revealed a (weaker) main effect of source credibility, β = 0.10, *t*(1,078.8) = 1.99, *p* = .047, *d* = 0.11. Assertions stated by a high-credible source (*M* = 4.06 *SE* = 0.13) led to slightly higher plausibility ratings than assertions stated by a low-credible source (*M* = 3.86, *SE* = 0.13). However, this main effect was qualified by a significant interaction of plausibility and source credibility as expected in Hypothesis 2, β = 0.26, *t*(1,079.4) = 4.99, *p* < .001 (see Fig. 1). When participants rated the plausibility of an assertion that was inconsistent with world knowledge, plausibility ratings were higher when this assertion was stated by a person with low expertise (*M* = 2.72, *SE* = 0.15) compared with the same assertion stated by a person with high expertise (*M* = 2.41, *SE* = 0.15), *t*(1,079) = −2.12, *p* = .035, *d* = −0.17. In contrast, when participants rated the plausibility of an assertion that was consistent with world-knowledge, plausibility ratings were higher when this assertion was stated by a person with high expertise (*M* = 5.72, *SE* = 0.15) compared with the same assertion stated by a person with low expertise (*M* = 5.00, *SE* = 0.15), *t*(1,079) = 4.94, *p* < .001, *d* = 0.39. Thus, participants considered the source credibility for their explicit evaluations of the plausibility of information. In particular, the consistency of source credibility and assertion plausibility seemed to matter, showing that evaluating plausibility explicitly involves discourse knowledge (i.e., source credibility) and world knowledge (i.e., world knowledge about facts presented in the assertions).

#### Source credibility ratings

**Fig. 2:**
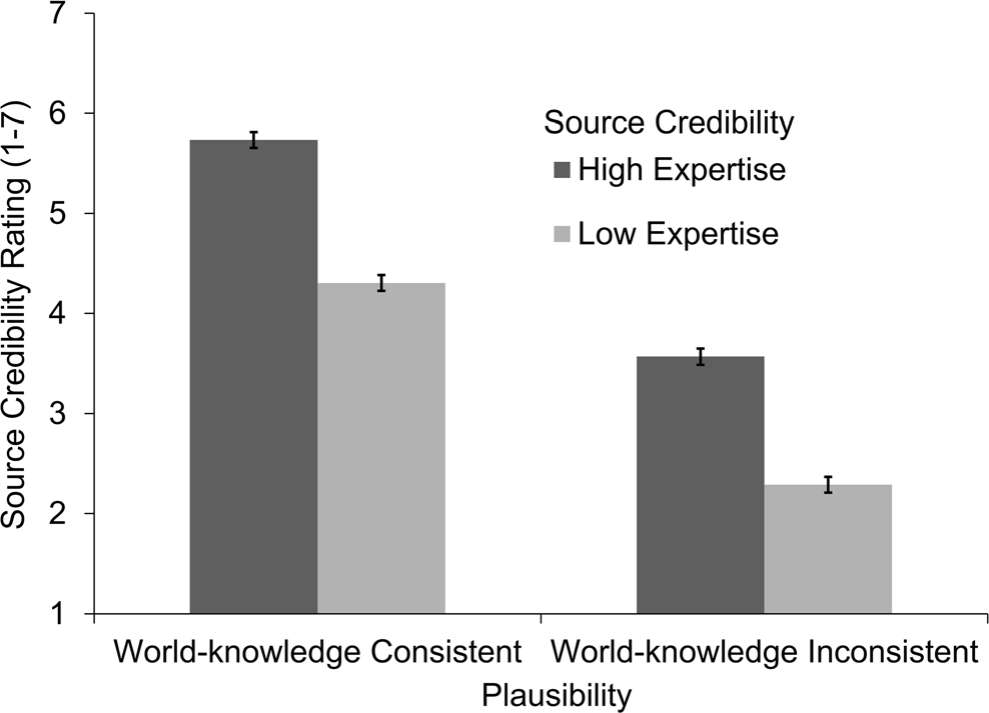
Mean source credibility ratings by experimental condition. Error bars represent the standard error of the mean

Source credibility ratings were available from 29 participants. We found significant main effects for both independent variables (see Fig. 2). As expected, the manipulation check confirmed the source credibility manipulation. Participants rated source credibility higher in stories with a high-expertise source (*M* = 4.65, *SE* = 0.14) compared with stories with a low-expertise source (*M* = 3.30 *SE* = 0.14), β = 0.68, *t*(906.3) = 13.27, *p* < .001, *d* = 0.79. Interestingly, stories with world-knowledge consistent assertions also led to higher ratings of source credibility (*M* = 5.02, *SE* = 0.14) than stories with world-knowledge inconsistent assertions, and the effect was even stronger (*M* =2.93 *SE* = 0.14), β = 1.05, *t*(902.8) = 20.49, *p* < .001, *d* = 1.22. The analysis revealed no significant interaction effect, β = 0.04, *t*(906.3) = 0.76, *p* = .448. In sum, persons with a high level of expertise in a certain field were rated more credible than persons with low expertise. Furthermore, participants seemed to take the plausibility of an assertion as an additional, if not to say the primary, source to evaluate source credibility regardless of expertise level (i.e., described source credibility). Participants possibly used relevant world knowledge to validate the stated assertions and used this comparison as a mean to evaluate source credibility.

#### Meaningfulness ratings

**Fig. 3:**
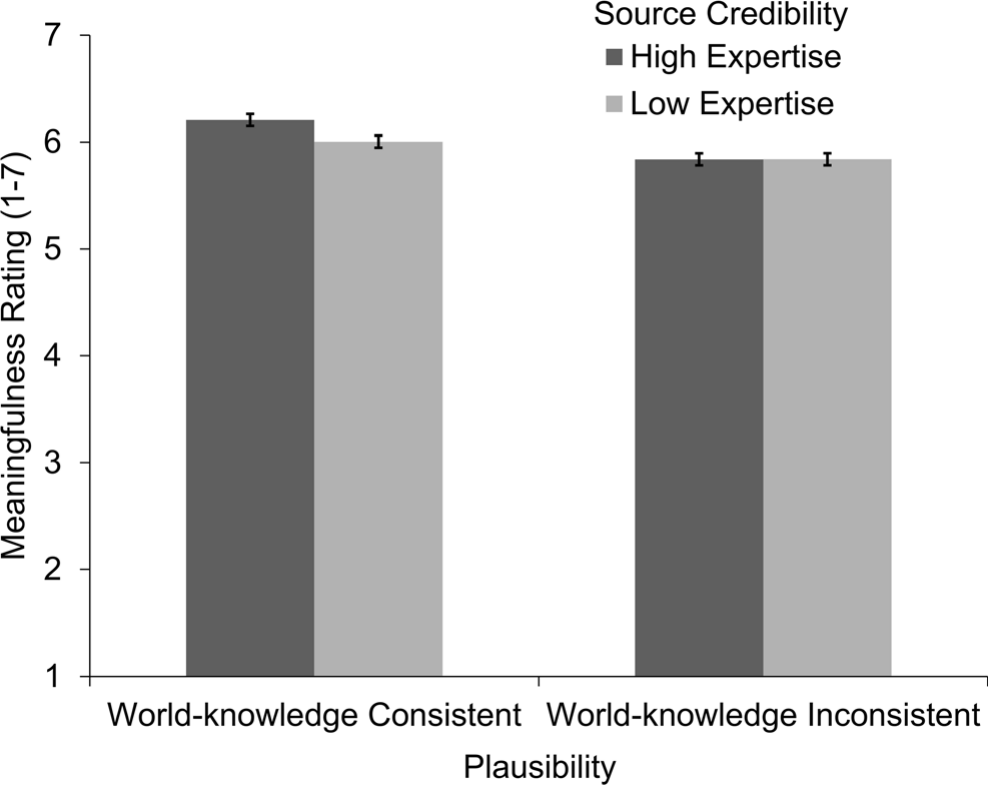
Mean meaningfulness ratings by experimental condition. Errors bars represent the standard error of the mean

Meaningfulness ratings were available from 63 participants. The results by experimental condition are displayed in Fig. 3. A significant effect of position was found, β = −0.04, *t*(2,038.5) = −2.08, *p* = .037. Participants rated stories presented in the beginning of the experiment slightly higher than stories presented later, possibly because of fatigue or boredom effects. Moreover, we found small main effects for both independent variables. Stories containing high-expertise sources (*M* = 6.02, *SE* = 0.11) led to higher meaningfulness ratings than low-expertise sources (*M* = 5.92, *SE* = 0.11), β = 0.05, *t*(2,007.1) = 2.60, *p* = .009, *d* = 0.08. In a similar pattern, stories with world-knowledge consistent assertions led to higher meaningfulness ratings (*M* = 6.11, *SE* = 0.11) than world-knowledge inconsistent assertions (*M* = 5.84, *SE* = 0.11), β = 0.13, *t*(2,006.8), *p* < .001, *d* = 0.22. Furthermore, a significant interaction effect was found, β = 0.05, *t*(2,006.4), *p* = .011. Stories with world-knowledge consistent assertions were rated as slightly more meaningful when the assertions were stated by a high-expertise source (*M* = 6.21, *SE* = 0.11) compared with a low-expertise source (*M* = 6.01, *SE* = 0.11), *t*(2,007) = 3.64, *p* < .001, *d* = 0.17. In world-knowledge inconsistent assertions, no difference was found in the meaningfulness ratings for stories containing a low credibility source and a high credibility source, *t*(2,007) = 0.03, *p* = .98. Thus, readers considered source credibility when required to rate the meaningfulness of stories that had a world-knowledge consistent assertion.

#### Comprehensibility ratings

**Fig. 4:**
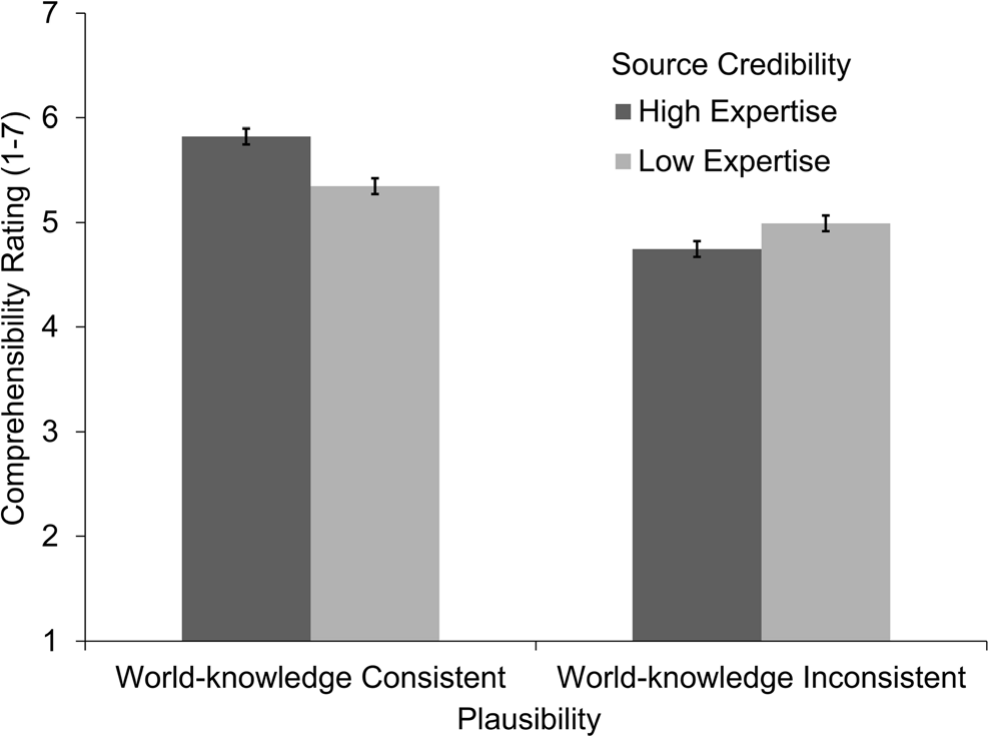
Mean comprehensibility ratings by experimental condition. Error bars represent the standard error of the mean

Comprehensibility ratings were available from 63 participants. The results by experimental condition are displayed in Fig. 4. The position of a story again had a significant effect on the ratings, β = −0.06, *t*(2,036.7) = -2.01, *p* = .044. Earlier stories led to higher comprehensibility ratings than later stories. Moreover, we found a significant main effect of source credibility, β = 0.06, *t*(2,003.4) = 2.00, *p* = .046, *d* = 0.08. Stories containing a high-expertise source (*M* = 5.29, *SE* = 0.14) led to slightly higher comprehensibility ratings than stories containing a low-expertise source (*M* = 5.17, *SE* = 0.14). We also found a significant main effect of plausibility, β = 0.36, *t*(2,002.9) = 12.23, *p* < .001, *d* = 0.58. Stories with a world-knowledge consistent assertion (*M* = 5.59, *SE* = 0.14) led to higher comprehensibility ratings than stories with a world-knowledge inconsistent assertion (*M* = 4.87, *SE* = 0.14). The analysis revealed a significant interaction effect, β = 0.18, *t*(2,002.5) = 6.19, *p* < .001. Again, stories with world-knowledge consistent assertions were rated as slightly more comprehensible when the assertions were stated by a high-expertise source (*M* = 5.82, *SE* = 0.15) compared with a low-expertise source (*M* = 5.35, *SE* = 0.15), *t*(2,003) = 5.79, *p* < .001, *d* = 0.39. In contrast, for stories with world-knowledge inconsistent assertions, high-expertise sources led to lower comprehensibility ratings (*M* = 4.75, *SE* = 0.15) than stories with low-expertise sources (*M* = 4.99, *SE* = 0.15), *t*(2,003) = −2.97, *p* = .003, *d* = −0.20.

In sum, the higher ratings for comprehensibility on consistent stories reflect to some extent the fit between source credibility and the plausibility of an assertion.

#### Plausibility ratings (comprehensibility and meaningfulness controlled for)

Given that the patterns of results obtained for comprehensibility and meaningfulness partially resembled the results obtained for the focal dependent variable plausibility, we reran the analyses controlling for comprehensibility and meaningfulness by including these ratings as centered predictors in the models. The model revealed a positive association of comprehensibility and plausibility ratings, β = 0.74, *t*(1,041) = 11.93, *p* < .001. Importantly, however, the effects relevant for the hypotheses—that is, the main effect of plausibility, β = 1.20, *t*(1,092) = 23.98, *p* < .001, *d* = 1.40, and the interaction of plausibility and source credibility, β = 0.18, *t*(1,074) = 3.73, *p* < .001—remained intact. These results suggest that the plausibility ratings are not identical with global comprehension but reflect judgments specific to validation.

In sum, the results of Experiment 1 show that readers evaluate and weigh source credibility in their explicit judgments of information plausibility. Apparently, the consistency of source information and plausibility matters. Credible sources boost the perceived plausibility of plausible information but lower the perceived plausibility of implausible information. A similar but less pronounced pattern was found for comprehensibility and to a smaller extent (and only for stories with world-knowledge inconsistent assertions) for meaningfulness ratings. Assuming that these ratings reflect metacognitive judgments of successful comprehension, our findings underscore the relevance of validation (as reflected in the plausibility ratings) for comprehension and the strong relationship between validation and integration (e.g., Richter, 2015).

Lastly, not only was perceived plausibility affected by source credibility, but plausibility also affected the perceived source credibility. This exploratory finding suggests that source credibility and plausibility might have a more dynamic relationship than commonly assumed.

Experiment 1 investigated off-line outcomes of validation by employing explicit measurements of plausibility and source credibility judgements. The findings of Experiment 1 are informative with regard to validation insofar as nonstrategic validation processes are assumed to feed into explicit plausibility judgments (e.g., Schroeder et al., 2008). However, the off-line judgements collected in Experiment 1 are also likely to involve reflective processes and are based in part on the global impression of the situation described in the story. Thus, to gain a clearer picture of the moment-to-moment processes involved in validation and the role of source credibility in these processes, we conducted Experiment 2, which included reading times as implicit indicators of validation.

## Experiment 2

Experiment 2 was highly similar to Experiment 1, but the dependent variables were reading times for the target sentences, which varied in plausibility, and reading times for spillover sentences (i.e., the sentences immediately following the target sentence). Reading times for spillover sentences were examined to shed light on the time course of using source credibility when forming plausibility judgments in the nonstrategic validation process as defined by the RI-Val model.

The general expectation was that the pattern of results for the reading times obtained in Experiment 2 would mirror the results found for the plausibility ratings in Experiment 1. Specifically, we expected readers to process assertions that are consistent with world knowledge faster than assertions that are inconsistent with world knowledge (Hypothesis 3a). Longer processing times for world-knowledge inconsistent assertions have been shown numerous times with the contradiction paradigm (see Cook & O’Brien, 2014, for an overview) and are usually interpreted as indicating the detection of the implausibility through validation. More importantly, however, we expected plausibility to interact with source credibility. A matching combination of source credibility and plausibility should lead to faster reading times because world knowledge and discourse knowledge align, allowing faster validation. In contrast, a mismatching or inconsistent combination of source credibility and plausibility should lead to slower reading times compared with the consistent combination. For example, the consistent combination of an expert on the topic of astronomy and stars (high-expertise source) stating that Jupiter is the biggest planet in the solar system (consistent with world knowledge) should lead to faster reading times than a low-expertise source stating a fact that is world-knowledge consistent. On the other hand, a matching combination of a nonexpert on the topic of astronomy and stars, stating that the sun is the biggest planet in the solar system, should lead to faster reading times compared with an expert stating a fact that is world knowledge inconsistent (Hypothesis 4a).

The RI-Val model (O’Brien & Cook, 2016) assumes that resonance, integration, and validation processes are asynchronous, parallel, and passive, and that they run to completion. In line with these critical assumptions, the expected effects on target sentences should also be revealed on the subsequent (i.e., spillover) sentences. Moreover, if the temporal assumptions of the RI-Val model hold, the effect on the spillover sentences might be even more pronounced than on the target sentence. We expected reading times of the spillover sentence to be slower for world-knowledge inconsistent compared with world-knowledge consistent assertions (Hypothesis 3b) and an interaction of plausibility and source credibility, with consistent combinations of source credibility and plausibility leading to faster reading (Hypothesis 4b). Given that such delayed effects are particular to validation (according to the RI-Val model) and not so much to integration or activation, this pattern of effects would specifically corroborate the general assumption that source credibility affects validation.

### Method

#### Participants

We recruited 68 participants with an average age of 25.75 years (*SD* = 7.68 years). Most participants were students from the University of Würzburg (82%) and female (75%). We used the online participant management software at the University of Würzburg (SONA Systems) to recruit participants. Four participants reported a first language other than German; one participant reported a language impairment. The data from these participants were excluded from the analyses. Participants received 7 euros for participation.

#### Materials

We selected 28 of the 36 stories from Experiment 1 for inclusion in Experiment 2. For all 28 stories, significant differences were found in plausibility ratings between the story versions with world-knowledge consistent versus world-knowledge inconsistent assertions and significant differences in source credibility ratings between the version with the high-credible and the low-credible sources. The length (mean number of characters) was comparable across the experimental story versions (high expertise–world-knowledge consistent assertion: *M* = 635.34, *SD* = 76.91; high expertise–world-knowledge inconsistent assertion: *M* = 633.79, *SD* = 76.37; low expertise–world-knowledge consistent assertion: *M* = 635.42, *SD* = 77.26; low expertise–world-knowledge inconsistent assertion: *M* = 634.63, *SD* = 78.17). On average, the experimental stories had a Flesch score (Flesch, 1948; German adaptation by Amstad, 1978) of 56.22 (*SD* = 5.83) comparable to Experiment 1. Thus, the stories were “demanding” or “fairly difficult to read.” We translated and adapted 20 filler stories from Foy et al. (2017). The filler stories consisted of eight sentences with topics and linguistic characteristics comparable to the experimental stories. The filler stories had no explicit descriptions of expertise and no direct speech. All filler stories were plausible.

#### Norming study

We conducted a (post hoc) norming study with the selected 28 stories from Experiment 1 (plus eight additional stories required for an independent study) to confirm that the high-credible and low-credible story versions differed in perceived credibility between the two sources. The participants (*N* = 48) were mostly female (87.5%) and undergraduates from the University of Würzburg and were reimbursed with 5 euros. The average age was 23.38 (*SD* = 6.27). Participants read the 36 stories in a randomized order and rated plausibility (1 = *very implausible* to 7 = *very plausible*) of the assertions and credibility of the introduced source (1 = *not credible at all* to 7 = *very credible*) with respect to the field of expertise associated with the assertion. Presentation of story versions and the order of the two rating tasks were counterbalanced across participants. High-expertise sources received significantly higher source credibility ratings (*M* = 4.56, *SE* = 0.15) than low-expertise sources (*M* = 3.29, *SE* = 0.15), β = 0.63, *t*(811.4) = 10.10, *p* < .001, *d* = 0.68.

#### Design

The design was a 2 (source credibility: high expertise versus low expertise) × 2 (plausibility: world-knowledge consistent versus world-knowledge inconsistent assertion) within-subjects design. Each participant read one version of every story. We counterbalanced the assignment of stories to experimental conditions across participants via a Latin square (four different lists). The dependent variable was reading time per sentence (in ms) for the target sentence and the subsequent sentence (spillover sentence). Each participant read the stories in a randomized order.

#### Procedure

Participants were tested in groups of up to eight people and gave informed consent. Their instruction was to read the stories for comprehension and to answer questions after some of the stories. The software Inquisit 5 (Version 5; Millisecond Software, Seattle, WA) was again used for presenting the stimuli and recording the dependent variables. Participants read all 48 stories on a computer screen in a self-paced manner identical to Experiment 1. Four practice trials were included at the beginning. After every filler story, participants responded to a yes/no comprehension question (e.g., “Was Maria prepared for her son’s birthday?”). The correct answer to half of the questions was yes. The experiment lasted approximately 30 min. Participants needed on average 25.37 min (*SD* = 6.06) to read all 48 stories.

### Results and discussion

In addition to the data obtained from the four nonnative speakers and the participant with a reported language impairment, data from two participants were excluded because a software error occurred during the experiment. Moreover, two participants with an accuracy below 70% in the comprehension questions were also excluded. The final sample consisted of 59 participants with a mean accuracy of 87.91% (*SD* = 7.53) on the comprehension questions. Reading times outside the interval defined by three standard deviations above or below the participant or item mean were treated as missing values (33 data points or 1.2% of the data points for target sentences, six data points or 0.3% for spillover sentences). Reading-time data of target sentences and spillover sentences were analyzed with linear mixed models with random effects (random intercepts) of participants and stories (see Tables 2 and 3). We entered main effects as well as the interaction of both factors as fixed effects in the model. Contrast-coding was used as in Experiment 1. Additionally, we entered sentence length and the position of the story in the experiment as centered predictors (fixed effects) to control for item length and position effects.

**Table 2.** Estimated coefficients, standard errors, degrees of freedom, and *t* values for the linear mixed model of the reading times of the target sentence in Experiment 2

	Est.	*SE*	*df*	*t*	
(Intercept)	3,556.64	133.14	76.97	26.71	***
Length of sentence	536.64	72.65	31.94	7.39	***
Position	−291.01	27.76	1,539.68	−10.48	***
Source credibility	30.48	27.30	1,529.27	−1.12	
Plausibility	−207.54	27.46	1,547.65	−7.56	***
Source Credibility × Plausibility	−60.85	27.29	1,528.95	−2.23	*

*Note.* Source credibility (contrast coded: high expertise = 1, low expertise = −1). Plausibility (contrast coded: world-knowledge consistent = 1, world-knowledge inconsistent = −1).

*p < .05, **p < .01, ***p < .001

**Table 3.** Estimated coefficients, standard errors, degrees of freedom, and *t* values for the linear mixed model of the reading times of the spillover sentence in Experiment 2

	Est*.*	*SE*	*df*	*t*	
(Intercept)	2,494.15	80.29	75.46	31.06	***
Length of sentence	455.91	42.05	25.92	10.84	***
Position	−198.43	18.03	1,554.35	−11.00	***
Source credibility	19.79	17.73	1,540.66	1.12	
Plausibility	−37.30	17.74	1,541.25	−2.10	*
Source Credibility × Plausibility	−58.27	17.73	1,540.59	−3.29	**

*Note.* Source credibility (contrast coded: high expertise = 1, low expertise = −1). Plausibility (contrast coded: world-knowledge consistent = 1, world-knowledge inconsistent = −1).

*p < .05, **p < .01, ***p < .001

#### Target sentences

**Fig. 5:**
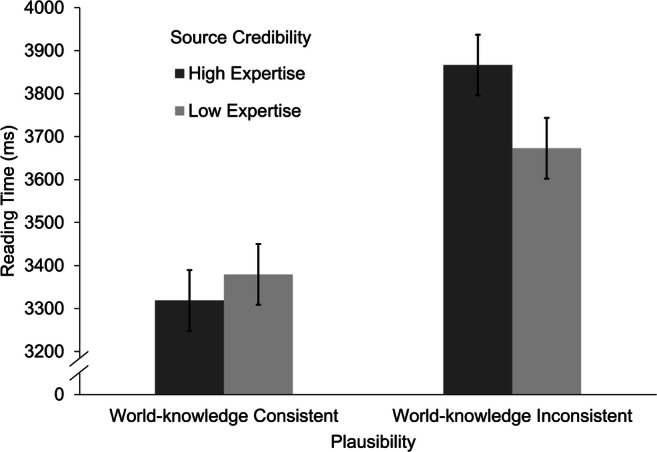
Mean reading times on target sentence by experimental condition. Error bars represent the standard error of the mean

The sentence length and the position of the story in the experiment had a significant effect on reading times. Longer target sentences led to slower reading times, β = 536.64, *t*(31.9) = 7.39, *p* < .001. Participants needed more time to read target sentences in stories presented earlier in the experiment, β = −291.01, *t*(1,539.7) = −10.48, *p* < .001. As predicted in Hypothesis 3a, we found a significant main effect of plausibility, β = −207.54, *t*(1,547.7) = −7.56, *p* < .001, *d* = −0.29. World-knowledge inconsistent sentences (*M* = 3764 ms, *SE* = 136 ms) were read more slowly than world-knowledge consistent sentences (*M* = 3349 ms, *SE* = 136 ms). More importantly, the interaction effect of plausibility and source credibility predicted in Hypothesis 4a emerged, β = −60.85, *t*(1,529) = −2.23, *p* = .026. The pattern of the interaction (see Fig. 5) partly mirrored the interaction found in Experiment 1 for the explicit plausibility ratings. Reading times for world-knowledge inconsistent sentences were slower when combined with a source with high (*M* = 3,856 ms, *SE* = 141 ms) compared with low credibility (*M* = 3,673 ms, *SE* = 141 ms), *t*(1,529) = 2.36, *p* = .019, *d* = 0.13. Reading times for target sentences that were consistent with world knowledge where slightly faster when combined with a source with high credibility (*M* = 3,319 ms, *SE* = 141 ms) compared with a source with low credibility (*M* = 3,379 ms, *SE* = 141 ms), but this difference was not significant, *t*(1,529) = −0.79, *p* = .42. These results provide partial support for Hypothesis 4a. Information about source credibility seems to modulate the nonstrategic validation of implausible information.

#### Spillover sentences

**Fig. 6:**
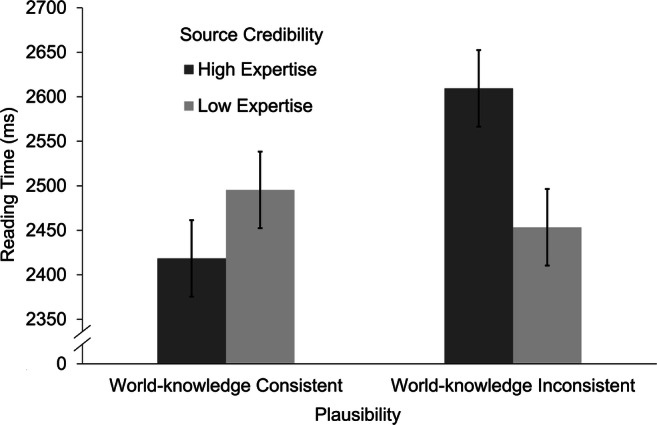
Mean reading times on spillover sentence by experimental condition. Error bars represent the standard error of the mean

We expected similar and potentially even more pronounced effects to occur for spillover sentences. Sentence length and position of the story in the experiment exerted significant effects on reading times. Longer spillover sentences led to higher reading times, β = 455.91, *t*(25.9) = 10.84, *p* < .001. The same was true of stories presented earlier in the experiment, β = −198.43, *t*(1,554.4) = −11.00, *p* < .001. In addition, the analysis revealed a significant main effect of plausibility (Hypothesis 3b), β = −37.30, *t*(1,541.3) = −2.10, *p* = .036, *d* = −0.08, and no main effect of source credibility, β = 19.79, *t*(1,540.7) = 2.12, *p* = .265. Spillover sentences subsequent to world-knowledge-inconsistent target sentences led to slower reading times (*M* = 2,531 ms, *SE* = 82 ms) than spillover sentences subsequent to world-knowledge-consistent target sentences (*M* = 2,457 ms, *SE* = 82 ms). More importantly, the expected interaction of source credibility and plausibility on reading times of the spillover sentences (Hypothesis 4b) was significant, β = −58.27, *t*(1,540.6) = −3.29, *p* = .001 (see Fig. 6). Reading times for spillover sentences following a world-knowledge-inconsistent target sentence were slower when combined with a source with high (*M* = 2,609 ms, *SE* = 86 ms) compared with low credibility (*M* = 2,453 ms, *SE* = 86 ms), *t*(1,541) = 3.11, *p* = .002, *d* = 0.17. In contrast, spillover sentences following a world-knowledge consistent target sentence combined with a high credibility source (*M* = 2,418 ms, *SE* = 86) led to faster reading times than spillover sentences following a world-knowledge consistent target sentence combined with a low credibility source (*M* = 2,610, *SE* = 86), but this difference failed to reach significance, *t*(1541) = −1.54, *p* = .124. Thus, Hypothesis 4b regarding the modulating role of source credibility for validation was again partially supported. Evidence was found for the claim that source credibility modulated the validation of implausible assertions.

#### Reading times for target and spillover sentences (controlling for mean comprehensibility and meaningfulness)

We reran the analyses controlling for the mean comprehensibility and meaningfulness ratings obtained for each story in Experiment 1 to assess the potential influence of these variables on the reading times for target and spillover sentences. The mean ratings were included as centered predictors in the models. The analyses provided no evidence for effects of comprehensibility and meaningfulness on the reading times for the target sentence or the spillover sentence (for all effects, *p* > .352). The effects relevant for the hypotheses remained largely intact. The main effect of plausibility on the reading times was still significant for the target sentence, β = −211.85, *t*(1,553.7) = −4.99, *p* < .001, *d* = −0.30, but not for the spillover sentence, β = −22.56, *t*(1,544.5) = −0.83, *p* = .410. Importantly, however, the interaction of plausibility and source credibility (predicted by Hypotheses 4a and 4b) was significant in the model for the target sentence, β = −66.47, *t*(1,561.4) = −2.06, *p* = .040, and in the model for the spillover sentence, β = −49.16, *t*(1,572.5) = −2.36, *p* = .019. These results suggest that the pattern of reading times, especially the focal interaction of source credibility and plausibility, cannot be explained by differences in perceived comprehensibility and meaningfulness between the stories.

The similar pattern of results for target and spillover sentences lends further support to the assumption that source credibility is used in the validation of information. Moreover, the fact that the pattern was even more pronounced for the spillover sentences is in line with the RI-Val model (O’Brien & Cook, 2016) that validation processes start later than (but parallel to) integration processes and run to completion. However, evidence for a modulating effect of source information on validation were found only for world-knowledge inconsistent sentences, where a low-expertise source reduced the slowdown in reading typically found for knowledge-inconsistent information.

## General discussion

The present experiments examined the possibility that world knowledge and source credibility jointly influence the validation of text information. Participants read short narratives with high-credible or low-credible sources that stated information that was consistent or inconsistent with world knowledge. In Experiment 1, we used plausibility and source credibility ratings as an explicit measurement of evaluation. In Experiment 2, we used reading times of the target and spillover sentences as an implicit online measurement of validation.

In line with our predictions, we found strong main effects of plausibility on the plausibility ratings (Hypothesis 1) and on reading times for the target sentences, whose plausibility was varied (Hypothesis 3a), and for the subsequent spillover sentence (Hypothesis 3b). Moreover, we found a significant interaction effect of source credibility and plausibility with both explicit and implicit measurements. In line with Hypothesis 2, participants rated world-knowledge inconsistent assertions as less plausible when the assertions came from a high-credible source compared with a low-credible source. Supporting Hypothesis 2 further, participants also rated world-knowledge consistent assertions as more plausible when the assertions came from a high-credible source compared with a low-credible source. Similarly, and in line with Hypotheses 4a and 4b, participants read the target and the subsequent spillover sentences more slowly when a high-credible source stated world-knowledge inconsistent information compared with a low-credible source stating this information. These findings provide evidence for a possible modulating effect of source credibility on validation for world-knowledge inconsistent sentences.

### The different roles of source information and world knowledge in validation

Together, Experiments 1 and 2 show a convergence of online and off-line indicators of validation for world-knowledge inconsistent sentences but not for world-knowledge consistent sentences. Assertions that were consistent with world knowledge were rated as less plausible when they came from a low-expertise source, but the expertise of the source did not affect moment-to-moment reading times of the target and the spillover sentence, which we interpret as indicators of validation during reading. On the one hand, the similar patterns of plausibility judgments and reading times for world-knowledge inconsistent information lend support to the conclusion that the slowdown in reading times reflects validation processes, in particular the (implicit) detection of inconsistencies of information with world knowledge and the current discourse context. On the other hand, the divergent results for the world-knowledge inconsistent information might be explained by different processing foci and processing modes, a more local and passive mode for the reading times and a more global and reflective mode for the plausibility judgements (for a similar line of reasoning, see Egidi & Gerrig, 2006; Foy & Gerrig, 2014; Rapp & Mensink, 2011; Sparks & Rapp, 2011). Apparently, source information is only considered in moment-by-moment validation processes when an inconsistency of text information and knowledge occurs. In other words, world-knowledge consistency is the primary criterion used in validation, and source information, as a special kind of contextual information, is considered only when validation has revealed an inconsistency. In terms of the RI-Val model (O’Brien & Cook, 2016), source information is a kind of text contextual information that potentially competes with world knowledge that is the primary source of validation. Apparently, the contextual influence of source information is not strong enough to overturn the influence of activated world knowledge when the text information is consistent with that knowledge. In this case, the coherence threshold is reached quickly and readers move on to the next sentence. However, source information can influence the validation process when there is a mismatch between world knowledge and text information. In that case, it takes longer to reach the coherence threshold, which enables source information to take effect. It must be noted that this interpretation in terms of the RI-Val model, plausible as it may be, is mostly speculative at this point. Further experiments with methods allowing a more fine-grained analyses of the time course of using source information (such as eye-tracking methods) might be helpful to elucidate these issues.

The effects on the spillover sentences are also consistent with the critical assumptions of the RI-Val model (O’Brien & Cook, 2016), which states that activation, integration, and validation are parallel and asynchronous processes that run to completion. In line with the model, the joint impact of plausibility and source information lingered even after readers had moved on to the spillover sentences, which were identical across story versions. The interaction of plausibility and source credibility even became clearer on the spillover sentences, which is well in line with the temporal assumptions of the RI-Val model and our basic assumption that source credibility is a contextual factor that modulates the validation of text information.

### Effects of source information on validation might depend on the degree of (im-) plausibility and semantic overlap

To our knowledge, Foy et al. (2017) is the only study that presented evidence for the combined effects of source credibility and plausibility on validation, with high-credible sources mitigating the disruptive effects of implausible assertions on the comprehension process. At first sight, these findings might seem inconsistent with our finding that high-credible sources boosted the implausibility of implausible information. However, a key to understanding the differences lies in the type of implausibility used in the experiments by Foy et al. and our experiments. Foy and colleagues used sentences that described improbable events (e.g., a protagonist seeing a wolf in the backyard) for which the truth value of the sentences could not be determined by participants. Participants were thus required to infer the probability of the situation described in the critical assertion (e.g., “there are wolves in the backyard”) based on contextual information (e.g., the conversation takes place at a party) and associated prior knowledge (e.g., schematic knowledge about typical parties). The more prominent role of contextual information might have prompted participants to rely more strongly on source credibility cues, which may be construed as a specific type of contextual information, for validating the assertion. In contrast, participants in our experiments read sentences that could be judged as true or false based on world knowledge. Therefore, high-credible sources were perceived as inconsistent with world-knowledge inconsistent assertions rather than boosting their plausibility.

Thus, the size and the direction of the interactive effect of plausibility and source credibility possibly depend on the role of contextual knowledge and the degree of plausibility. According to social judgment theory (e.g., Sherif & Sherif, 1967; Sherif, Sherif, & Nebergall, 1965), judgments of belief-relevant information occur on a continuum with latitudes of acceptance, rejection, and noncommitment. A similar continuum might hold for validation, and source information would be most relevant for the validation of information that falls into the area of noncommitment, which implies uncertainty. To directly test this assumption, future research should vary plausibility within the same experiment—for example, with assertions gradually varying in plausibility. We expect that with increasing plausibility, the influence of source credibility would decrease and that credible sources would mitigate the disrupting effects of implausible assertions, but only up to a certain degree of implausibility. When the implausibility exceeds this threshold (as was presumably the case in the implausible statements used in our experiments), credible sources would increase the disrupting effect of implausible information. Preliminary evidence for the fruitfulness of this approach comes from Foy et al. (2017, Experiment 3). This experiment investigated the possible impact of varying plausibility on source credibility using narratives with plausibility-manipulated endings while having a low-credibility narrator in all conditions. Additionally, a high-credible source within the story gave affirming or contradicting information on the narrator’s perspective. Participants judged plausible story endings as significantly more plausible compared with implausible story endings. Notably, the plausible ending was judged as even more believable when a high-credible source (i.e., the police) confirmed the events, and thus plausibility was boosted by high-credible sources.

Moreover, a possible explanation for the different result pattern on spillover sentences between Foy et al. (2017) and our findings might be the degree of semantic overlap in the experimental texts. The texts used by Foy and colleagues consisted of a story continuation that affirmed or contradicted the plausible or implausible assertion, which induced high semantic overlap between the continuation and the assertion. Our experimental texts had less semantic overlap and thus might have caused a more delayed comprehension because the relevant background information (i.e., information about source credibility) needed more time to become activated and integrated. The higher semantic overlap in the stories of Foy et al. (2017) might have induced readers to require more integration of contextual information, as in verifying the assertion by accessing discourse knowledge, compared with our experiments in which readers could validate the assertions by accessing their world knowledge.

### Different dimensions of source credibility and their impact on validation

Another difference between our experiments and the experiments by Foy et al. (2017) is the way that source credibility was manipulated. Source credibility is commonly conceptualized with the two dimensions of expertise and trustworthiness (Self, 2009). Foy and colleagues varied trustworthiness. For example, low-credible sources in their experimental texts were on drugs, paranoid, or had other severe impairments of perception. In our experiments, we varied expertise through descriptions of the source’s occupations or education. Low-credible sources were described as persons with no knowledge about a specific topic, and high-credible sources were described as persons with very much knowledge about a specific topic or as experts in the specific topic (e.g., a university professor). Research on source credibility indicates that expertise and trustworthiness might elicit different effects on believability (see Pornpitakpan, 2004, for an overview), but findings are inconclusive. Nonetheless, these differences might play a role in explaining how source credibility is used in validation. One possibility is that varying a source’s expertise might be a less explicit manipulation than varying a person's trustworthiness by describing their general mental state as was done by Foy et al. (2017). However, the strong main effect of source credibility in the source-credibility ratings suggest that the expertise manipulation was effective.

### Validation and source information from the perspective of the D-ISC assumption

The D-ISC assumption (Braasch et al., 2012) states that readers are more likely to focus on source information when confronted with inconsistent information. As the findings by Braasch et al. (2012) and associated research (e.g., de Pereyra et al., 2014; Rouet et al., 2016; Saux et al., 2018) indicate, one way for readers to resolve the inconsistencies is to revisit the passage of the text with the source information or to provide more resources when initially processing the source information. In Experiment 2, we could not explore this possibility because the self-paced reading paradigm used in our experiments prevented readers from returning to previously read sentences. Even though comprehension is only marginally impaired by a self-paced reading paradigm with linear reading (Chung-Fat-Yim, Peterson & Mar, 2017), using other more naturalistic paradigms such as eye-tracking, which allow readers to regress to earlier sentences, would be fruitful for future research. In line with the D-ISC assumption (Braasch et al., 2012), we expect that readers who are confronted with inconsistencies, (e.g., a high-expertise source providing a false statement) would revisit the sentences that conveyed the source information in an attempt to reconcile the inconsistency.

An exploratory finding of Experiment 1 that might be relevant for the D-ISC assumption and related research is that the results suggest a more dynamic relationship between source credibility and plausibility than commonly assumed. Source credibility influenced perceived plausibility, but plausibility also influenced the perceived source credibility. Further research could focus more on this exploratory finding and attempt to disentangle the dynamic relationship of plausibility and source credibility.

### Further limitations and directions for future research

Future research should also provide participants with a clear definition of plausibility to assure participants have the same concept of plausibility in mind. Given the narrative context of the experimental stories and the possible story world that this context could induce, readers could have assessed plausibility differently than with other types of texts. Another limitation of our experiments might be the length and the repetitive character of the study. Reading 36 or even 48 stories consecutively might cause familiarity effects or even induce strategic processing. Some indication of position effects was found in the meaningfulness and comprehensibility ratings in Experiment 1 (which slightly decreased over the course of the experiment) and in the reading times in Experiment 2 (which also slightly decreased over the course of the experiment). Importantly, however, additional analyses (not reported here) revealed no indication that the hypothesized effects were moderated by the position of a story in the experiment.

### Conclusion

In conclusion, the present experiments provide further evidence for validation as a mechanism to maintain a coherent situation model. Our findings expand the emerging body of evidence from studies investigating possible conditions that influence validation—that is, the competition of contextual information and world knowledge and their impact on the component processes of comprehension as outlined in the RI-Val model (O’Brien & Cook, 2016). Apparently, source credibility can affect the validation of text information. Further research should map out the conditions that shape the interaction of plausibility and source information.

#### Author note

We would like to thank Sarah Engel, Laura Christin Katz, and Anna Plate for their help in constructing stimulus texts and collecting data. The experimental texts, data files, and R scripts for the full analyses are available (https://osf.io/3bcke/). The reported experiments were not preregistered. The authors report no conflict of interest.
